# Construction of Eight-Membered Carbocycles with Trisubstituted Double Bonds Using the Ring Closing Metathesis Reaction

**DOI:** 10.3390/molecules15064242

**Published:** 2010-06-11

**Authors:** Motoo Tori, Reiko Mizutani

**Affiliations:** Faculty of Pharmaceutical Sciences, Tokushima Bunri University, Yamashiro-cho, Tokushima, 770-8514, Japan

**Keywords:** ring closing metathesis, eight-membered carbocycles, synthesis

## Abstract

Medium sized carbocycles are particularly difficult to synthesize. Ring closing metathesis reactions (RCM) have recently been applied to construct eight-membered carbocycles, but trisubstituted double bonds in the eight-membered rings are more difficult to produce using RCM reactions. In this review, model examples and our own results are cited and the importance of the preparation of suitably designed precursors is discussed. Examples of RCM reactions used in the total synthesis of natural products are also outlined.

## 1. Introduction

**Figure 1:**
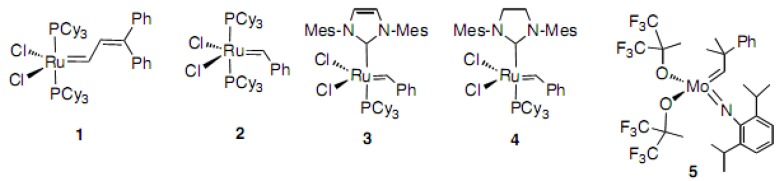
Structures of the reagents for RCM reactions.

Grubbs and his group developed ruthenium catalyst **1** in 1992 [1] and applied it to synthetic efforts [2,3]. Soon after, the first generation Grubbs catalyst **2** (Figure 1) was invented and became commercially available [4,5]. Molybdenum complex **5** (Schrock reagent) is known to work for the construction of trisubstituted double bonds; however, the complex is not so easy to handle [6,7]. Grubbs and his group further reported the commercially available so-called second-generation Grubbs catalyst **4** in 1999 [8]. Since then, ring closing metathesis (RCM) reactions have been used extensively by scientists, even in total syntheses [9,10,11,12,13,14,15]. The application of these catalysts began in the area of macrocyclic compounds, and later syntheses of more difficult 7-, 8-, 9- and 10-membered rings appeared in the literature [9,10]. In earlier work, RCM reactions were used in the synthesis of mostly heterocyclic compounds. We have been interested in these types of reactions, because terpenoids usually have trisubstituted double bonds in the carbocycles. However, the first generation Grubbs catalyst **2** was not applicable to the construction of the trisubstituted double bonds, but the second generation Grubbs catalyst **4** or imidazolinylidene catalyst **3** [16,17] can be used for such reactions. Thus, many chemists, as well as our group, started to use this commercially available catalyst. In this review, the synthesis of eight-membered carbocycles having disubstituted and the more difficult trisubstituted double bonds using RCM reactions will be presented [11,12,13,14]. A valuable review of enyne metathesis has appeared in the literature and therefore such syntheses are not included in this review [15].

## 2. Results and Discussion

### 2.1. Eight-membered carbocycles using RCM reactions (disubstituted double bonds)

It is well known that compounds possessing medium sized rings are difficult to prepare by conventional methods. Therefore, we have performed a preliminary check of the scope and limitations of the use of the Grubbs catalyst **2** for cyclization (Table 1). Cyclohexene **10b** was obtained quantitatively and cycloheptene **11b** was obtained in 40% yield, whereas cyclooctene **12b** was procduced in 14% and cyclononene **13b** in 4% yield, respectively. The larger ring compounds were not produced under these reaction conditions [18].

**Scheme 1:**
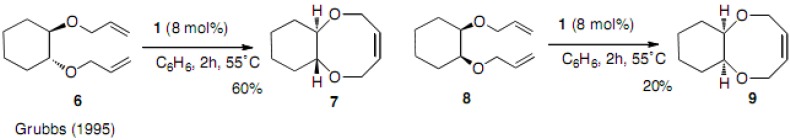
Cyclization to the dioxacyclooctene ring.

Grubbs reported the first application of his catalyst to the synthesis of eight-membered carbocycles using catalyst **1** (Scheme 1) [19]. Diallyl ether isomers had different reactivities towards the catalyst. The *trans* isomer afforded cyclooctene **7** in 60% yield, whereas a yield of 20% was afforded with the *cis* isomer **9**. Both the substituents of the *trans* isomer **6** can adopt equatorial positions, but in the case of the *cis* isomer **8**, one of them must be in the axial orientation.

**Table 1:** Cyclization to 6- to 11-membered compounds using catalyst **2** or **5**.

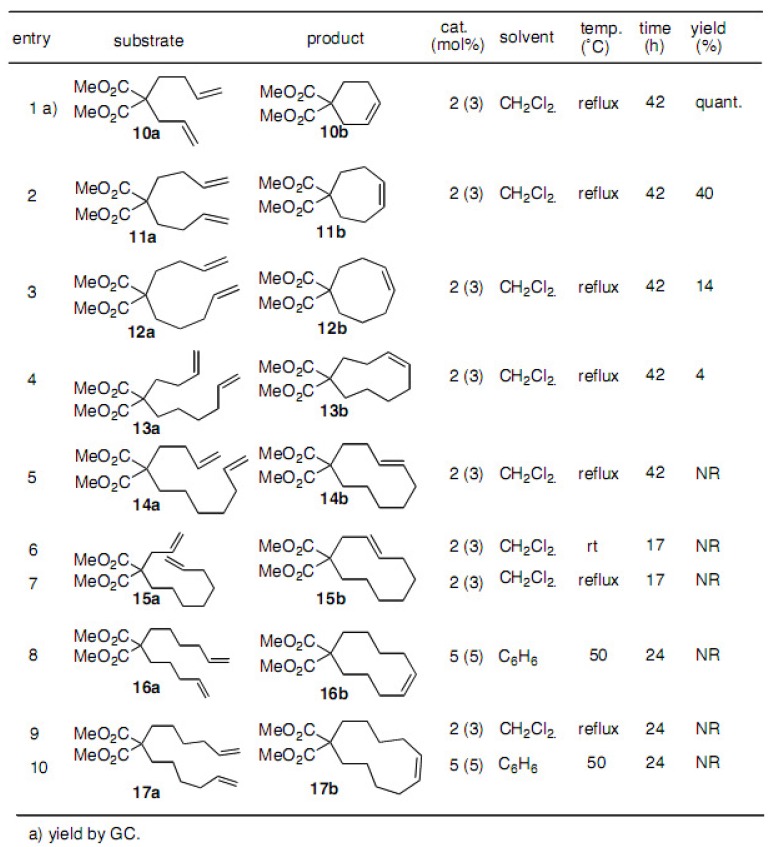

**Scheme 2:**
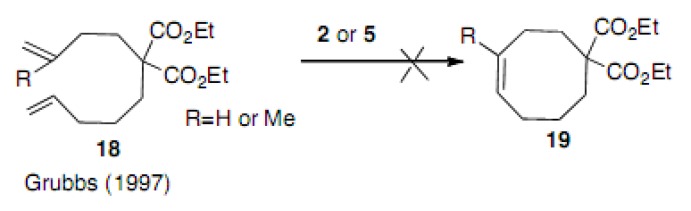
Attempt to cyclize a simple diene.

The application of the first generation Grubbs catalyst **2** to the cyclization reaction of diene compound **18** was reported. The first generation catalyst **2** or Schrock catalyst **5** did not work in this type of reaction with or without the methyl group (Scheme 2) [20]. This substrate **18** (R=H) differs from **12a** in its ester part. Grubbs did not describe the reaction conditions in detail. Successful cyclization (Table 1, entry 3) may be due to the longer reaction time. Although the Thorpe-Ingold effect is acting, it is not large enough to accelerate complete cyclization.

**Scheme 3:**
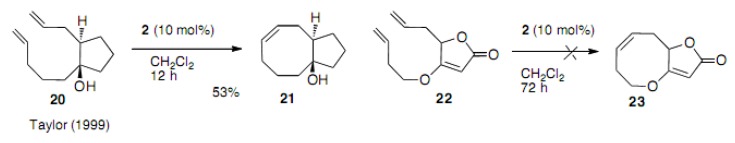
Attempts to cyclize fused systems.

An attempt by Taylor was interesting (Scheme 3) [21]. The diene alcohol **20** fused with the five membered ring cyclized to cyclooctene compound **21** in 53% yield. However, the enol-lactone **22** failed to cyclize. The reason why this compound is not a good substrate is unclear.

**Scheme 4:**
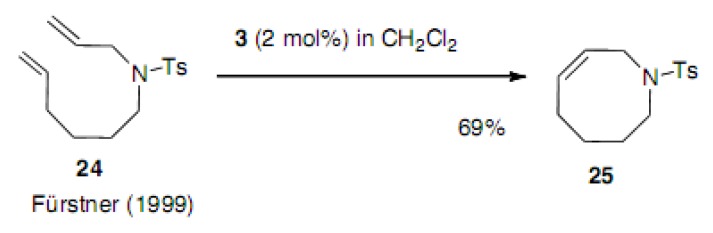
Formation of azacyclooctene.

Fürstner reported the successful cyclization of **24** to azacyclooctene **25** using Nolan catalyst **3** (Scheme 4) [22]. The power of some catalysts was compared in this report.

**Scheme 5:**
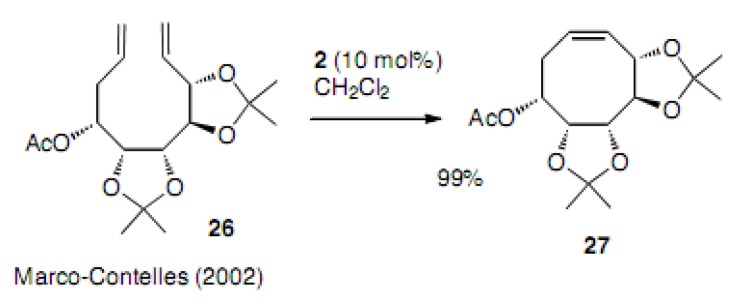
Cyclization to polyfunctional cyclooctene.

Construction of polyfunctional carbocycles were reported in 2002 (Scheme 5) [23]. Diene **26** bearing five functional oxygens produced the corresponding cyclooctene derivative **27** in 99% yield. It was a surprisingly high yield in the case of the first generation Grubbs catalyst **2**.

**Scheme 6:**
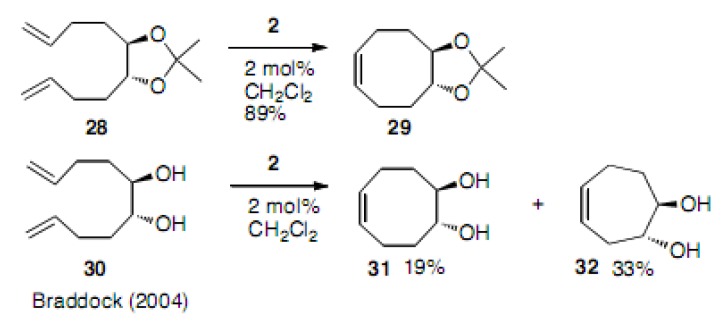
Cyclization to cyclooctene and cycloheptene.

Braddock reported that symmetrical diene **28** with a ketal protection cyclized to cyclooctene **29** in 89% yield, a comparable result to that shown in Scheme 5 [24]. However, the free substrate **30** produced only 19% yield of cyclooctene **31**, as well as the one-carbon less cycloheptene derivative **32** in 33% yield. This result apparently arose from the isomerization of the double bond into the more substituted position followed by cyclization to cycloheptene. The researchers noticed that in some cases the double bonds can isomerize and such a phenomenon occurs. Moreover, at higher temperatures more isomerization was observed to occur (Scheme 6). This is discussed later in this review.

**Scheme 7:**
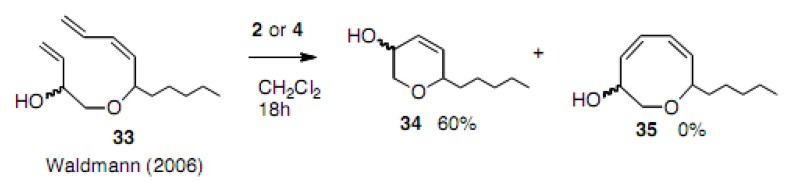
Competition to oxacyclooctadiene and oxacyclohexene.

Waldmann carried out an interesting experiment involving the competing cyclization to 6- or 8-membered compounds [25]. However, no cyclooctadiene **35** was obtained; instead cyclohexene **34** was formed in 60% yield (Scheme 7).

**Scheme 8:**
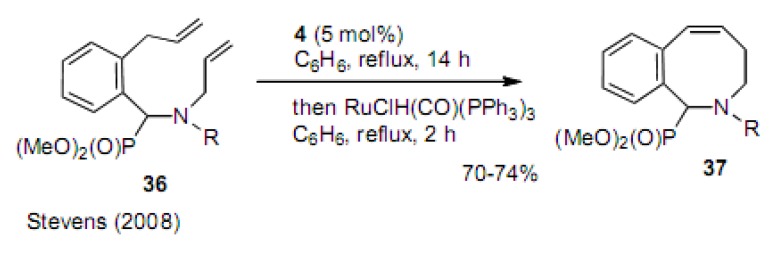
Cyclization of diene to benzoazacyclooctene.

As shown in Scheme 8, the double bond formed by the RCM reaction was isomerized to the conjugated position using catalyst **4**. Therefore, they used RuClH(CO)(PPh_3_)_3_ (2%) as an isomerization catalyst after the RCM reaction to produce **37** in 70−74% yield [26]. 

**Scheme 9:**
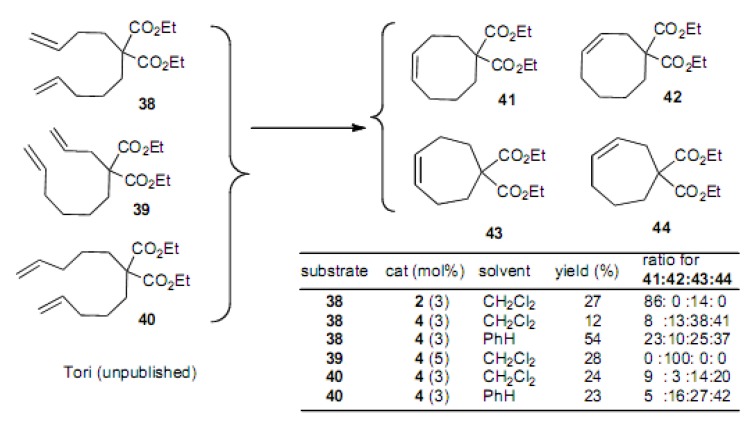
Attempted cyclization of simple dienes to 7-, 8- and 9-membered carbocycles.

We have been studying the scope and limitations of the RCM reaction using simple dienes, as shown in Scheme 9 [27]. In this systematic work, we have found that the isomerization of the double bond occurs in the RCM reaction in the case of the second generation Grubbs catalyst **4** and at higher temperatures. Thus, compound **38** afforded a mixture of compounds **41** and **43**, in the ratio of 86:14 with the first generation Grubbs catalyst **2**. However, with the second generation Grubbs catalyst **4** the same substrate produced a 12% yield of a mixture of **41**, **42**, **43** and **44**, in the ratio of 8:13:38:41. Interestingly, compound **39** afforded only compound **42** in 28% yield. Finally, compound **40** afforded a mixture of **41**, **42**, **43** and **44**, and no cyclononene derivative was obtained.

**Scheme 10:**
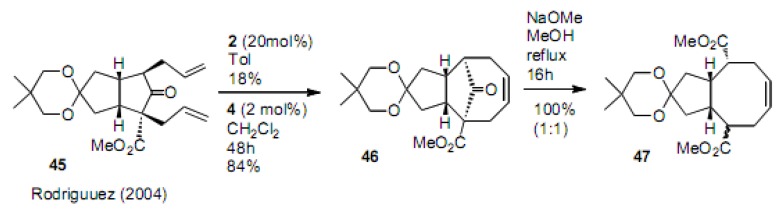
Tandem RCM to 7-membered carbocycle and ring opening to fused cyclooctene.

Further applications involving cyclization to cycloheptene derivative **46** and the ring opening to cyclooctene **47** was realized by Rodriguez (Scheme 10) [28,29]. An interesting review was published by Rodriguez [30].

**Scheme 11:**
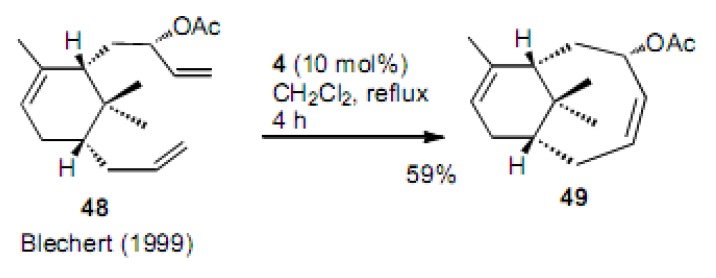
Cyclization to cyclooctene derivative, a part of the taxane skeleton.

A model study of the taxane skeleton was carried out for compound **48** (Scheme 11) [31]. Bicyclic acetate **49** was produced in 59% yield by the second generation Grubbs catalyst **4**. The acetoxy group diastereoisomer did not cyclize, but rather afforded dimeric compounds. This is clearly due to the steric hindrance of the *gem*-dimethyl groups present in the cyclohexane ring.

**Scheme 12:**
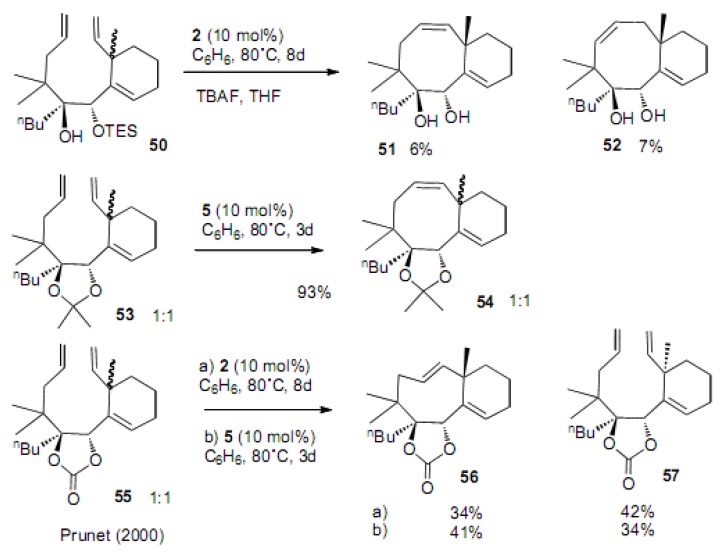
Effect of the protecting group in the cyclization to cyclooctene.

Prunet reported cyclization to fused cyclooctene systems (Scheme 12) [32,33]. The ease of cyclization was found to be strongly dependent on the protecting group. Mono TES protection of the diol **50** afforded only 6 and 7% yields of the corresponding diol **51** and its double bond isomer **52**, respectively. However, the ketal **54** was formed in 93% yield using the molybdenum catalyst **5**. Cyclic carbonate **55** produced the *trans* cyclooctene **56** for the first time in 30−40% yield with catalysts **2** or **5**. The product was carefully analyzed and the cyclized compound had *trans* stereochemistry regarding the methyl group at the juncture position and the butyl group. The recovered substrate **57** had *cis* stereochemistry. These examples clearly indicate the importance of the conformation of the substrates; however, it is unclear at this stage what kind of conformation these substrates adopt. Nonetheless, it is noteworthy that ketal or cyclic carbonate forced both the double bond moieties close to each other, and was formally recognized as a type of Thorpe-Ingold effect. It remains a mystery as to why the *trans* cyclooctene **56** survived under these reaction conditions, although it isomerized to *cis* cyclooctene under the similar reaction conditions.

**Scheme 13:**
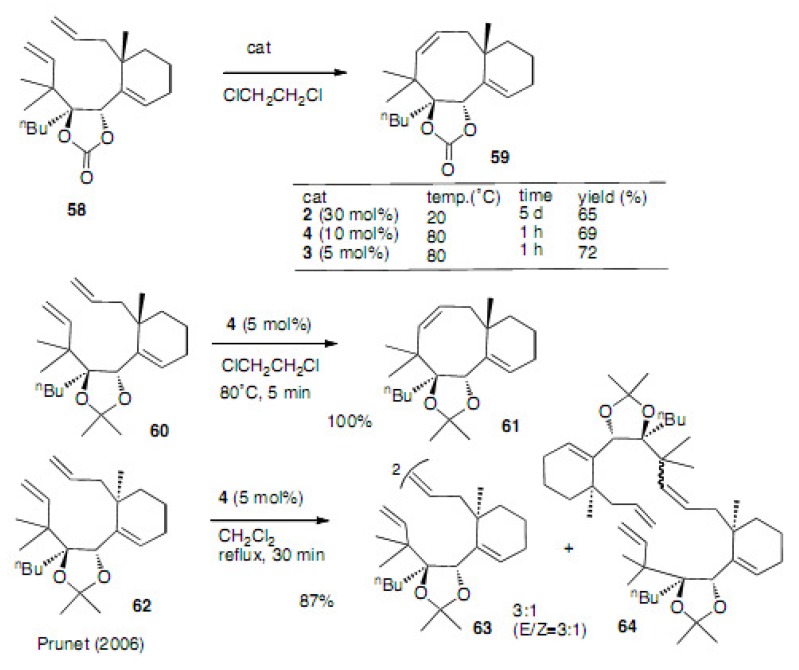
Comparison of the catalysts and the solvents.

After the recognition of the double bond isomer **52** in Scheme 12, Prunet changed the substrate to compound **58** with the proper stereochemistry (Scheme 13) [34,35]. Thus, by use of catalyst **2**, **3** and **4**, the desired compound **59** was isolated in 65, 72 and 69% yields, respectively. The use of dichloroethane as a solvent gave good results. Ketal instead of cyclic carbonate afforded a quantitative result with this solvent. Compound **62**, which bears the wrong stereochemistry at C8 for taxol, does not cyclize, even at higher temperatures.

### 2.2. Olefin rearrangement

**Scheme 14:**
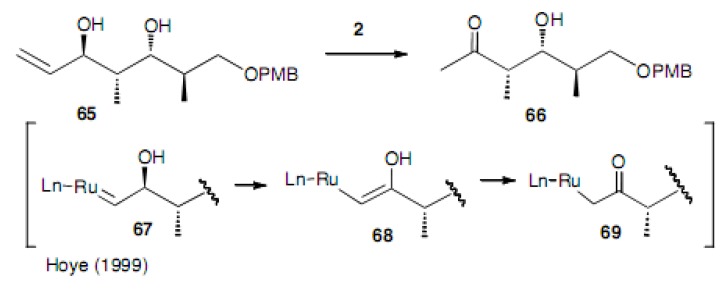
Olefin isomerization and the formation of a one-carbon less ketone.

Olefin isomerization under the RCM reaction conditions is discussed briefly. Hoye noticed that olefin isomerization occurred with Grubbs catalyst **2** to give one-carbon less ketone **66** and proposed a mechanism for this reaction (Scheme 14) [36]. 

**Scheme 15:**
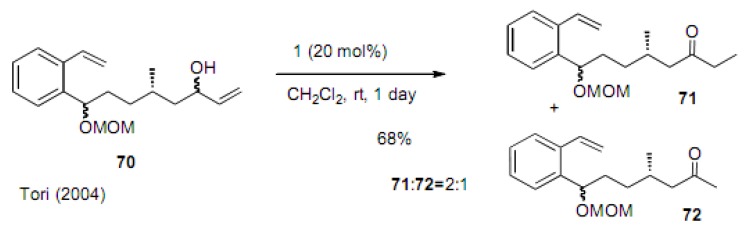
Olefin isomerization and the formation of two ketones.

Tori found that a similar isomerization and one-carbon less ketone **72** was formed in the reaction of compound **70** (Scheme 15) [37]. However, in addition to this ketone **72**, ethyl ketone **71** was also produced in the ratio of **71**:**72** = 2:1. This process just involves a rearrangement of the double bond to the inner position and the tautomerization to the ketone.

**Scheme 16:**
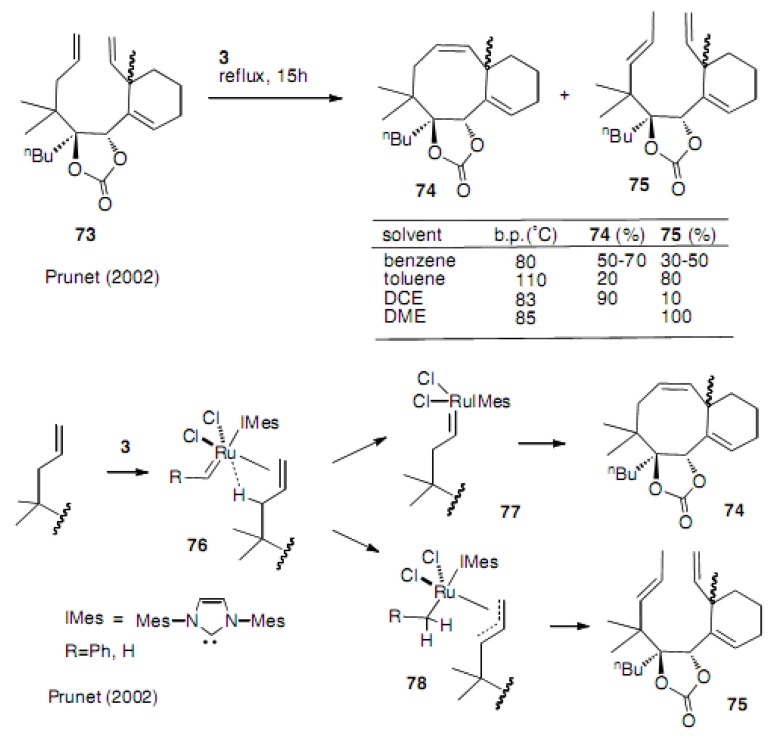
Olefin isomerization and the proposed mechanism.

Prunet and Nolan observed the isomerization of the double bond in a series of synthetic works on the taxane skeleton [38]. As shown in Scheme 16, with catalyst **3 **the more substituted olefin **75** was isolated at a higher temperature. The proposed mechanism involved the formation of the π-allyl complex **78** (oxidative addition) followed by hydride transfer to **75** (reductive elimination).

The report by Wagener in 2003 proposes hydride and allyl mechanisms [39]. A model study was carried out to see how much isomerization occurs under metathesis conditions. The second generation Grubbs catalysis **4** induces isomerization at 50−60 ºC under concentrated conditions. Schmidt reported the possibility of poarticipation of a ruthenium hydride complex in the olefin isomerization [40].

### 2.3. Eight-membered carbocycles using the RCM reaction (trisubstituted double bond)

**Scheme 17:**
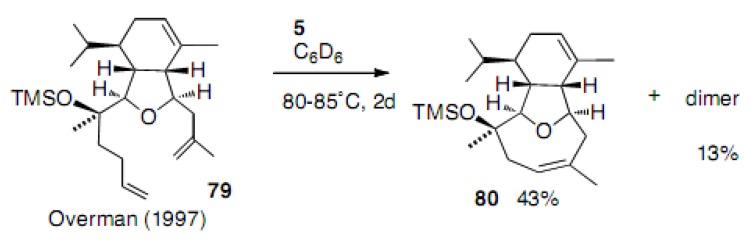
Formation of the one-carbon less carbocycle with a trisubstituted double bond.

Until now, formation of the cyclooctene ring with disubstituted double bonds was discussed. Hereafter trisubstituted double bonds in cyclooctene will be discussed. Overman aimed at constructing a nine-membered heterocycle, most likely carried out in an NMR tube in C_6_D_6_. Surprisingly, the product obtained in 43% yield was the eight-membered compound **80** (Scheme 17) [41]. This, of course, arose from the isomerization of the vinyl group into the inner double bond followed by the RCM reaction. In other words, the cyclooctene ring is easier to prepare than the cyclononene ring presumably due to steric hindrance. The difference in steric energy was calculated by CONFLEX [42,43] and discussed later (*vide infra*).

**Scheme 18:**
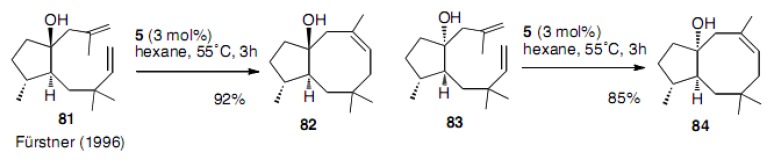
Synthesis of cyclooctene with a trisubstituted double bond.

Fürstner succeeded with the RCM reaction to afford both diastereoisomers **82** and **84** in high yields using the molybdenum catalyst **5** (Scheme 18) [44]. Very interestingly, hexane was used as the solvent. This was the first report describing the production of an eight-membered carbocycle with a trisubstituted double bond applied to the synthesis of the terpenoids.

**Scheme 19:**
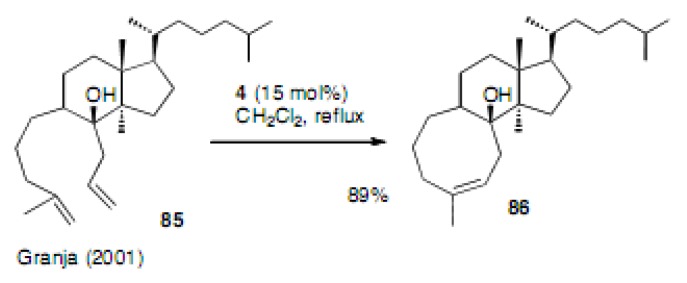
Cyclooctene in the steroid-like ring system.

The second generation Grubbs catalyst **4** was used for the construction of the trisubstituted cyclooctene ring by Granja (Scheme 19) [45]. The yield of **86** was 89% and this was a similar substrate to that employed by Fürstner.

**Scheme 20:**
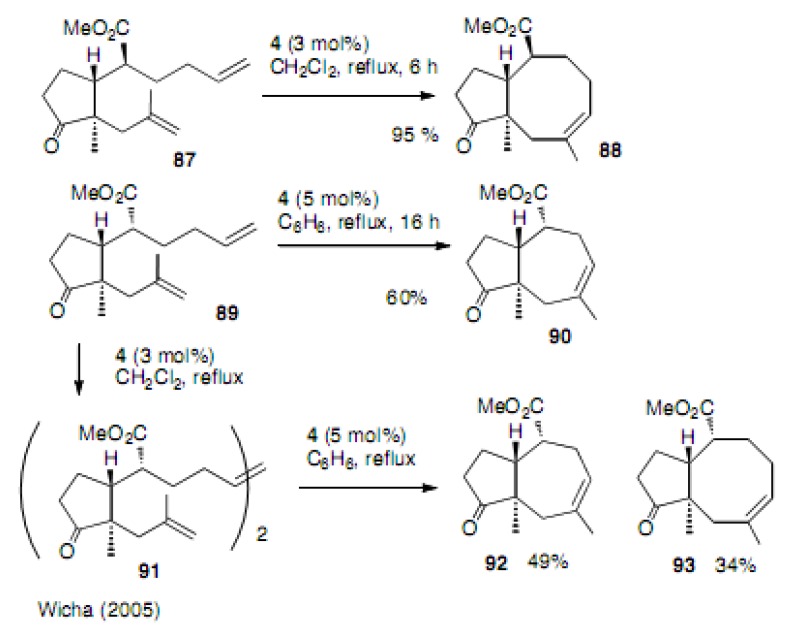
Tri-substituted double bond in the cyclooctene ring.

Wicha’s report is very interesting. Compound **87** was successfully cyclized to trisubstituted cyclooctene **88** in 95% yield (Scheme 20) [46]. However, its diastereoisomer **89** did not give cyclooctene, but rather cycloheptene **90**. The formation of the dimer **91** is a rapid process in CH_2_Cl_2_ and the reaction of this dimer with the same catalyst **4** in benzene affords both cyclooctene **93** and cycloheptene **92**. Once more, rearrangement occurred at a higher temperature. Wicha has presented an excellent review that includes their results [47].

**Scheme 21:**
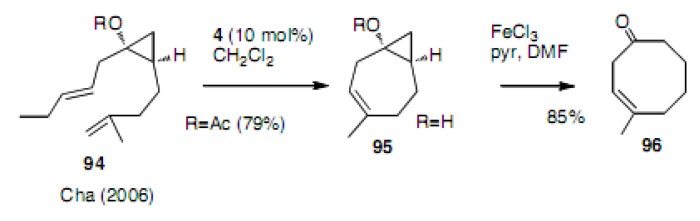
Tandem RCM and the ring opening of a three-membered ring.

An indirect approach to an eight-membered carbocycle has also been reported. For example, cycloheptene **95** was prepared by the RCM reaction followed by ring opening of the cylopropane ring to afford cyclooctenone **96** (Scheme 21) [48].

**Scheme 22:**
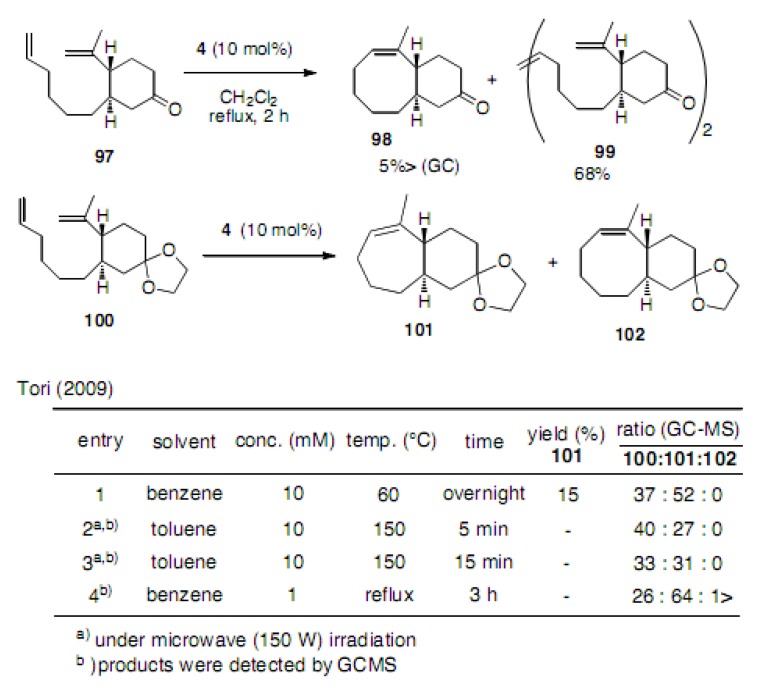
Attempts to cyclize to cyclooctene with a tri-substituted double bond.

A model study to produce eight-membered carbocycles was reported by Tori (Scheme 22) [49]. Compound **97** afforded only a trace amount of cyclooctene **98**. The main product was dimer **99** obtained in 68% yield. In the case of compound **100** almost no cyclooctene **102** was produced, instead cycloheptene **101** was isolated in 27−64% yield, depending on the temperature and the reaction time.

**Scheme 23:**
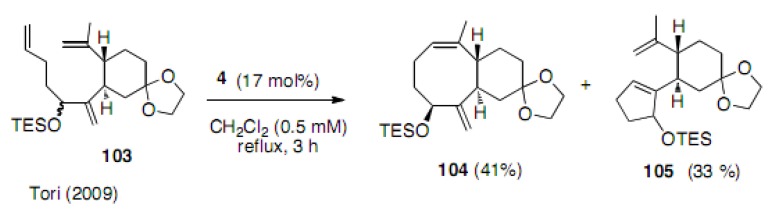
Attempts to synthesize cyclooctene.

Since the cyclization of compound **97** and **100** did not give good results. The sp^2^ carbon was introduced next to the cyclohexane ring to force two diene partners close to each other. The formation of the cyclooctene ring **104** was realized in 41% yield; however, the competitive cyclization to the five membered ring **105** occurred in 33% yield. Interestingly, the isolated compound **104** had the OTES group in the β orientation. However, the configuration of the five membered compound **105** was unclear, although it was a single product (Scheme 23) [49].

**Scheme 24:**
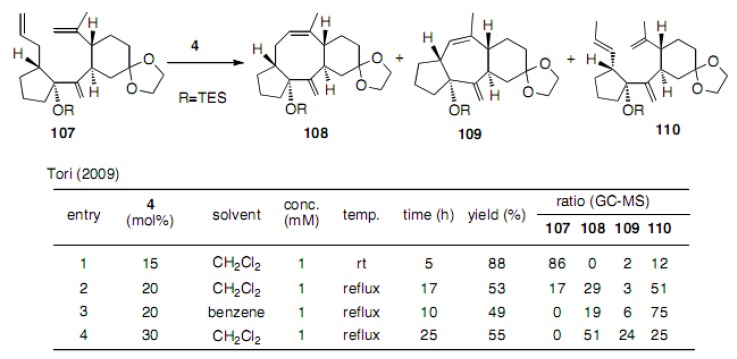
The effects of the configuration of substrates.

Further work toward the synthesis of sesterterpene YW3699 was described. Substrate **107** afforded three products **108**−**110** as shown in Scheme 24 [49]. Here, a normal RCM led to the formation of cyclooctene **108** and one-carbon less cycloheptene **109**, as well as isomerized olefin **110**.

**Scheme 25:**
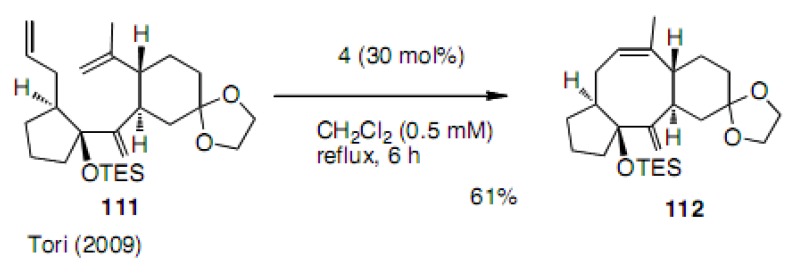
The effect of the configuration of substrates.

It is very interesting to note that the diastereoisomer **111** cleanly cyclized to cyclooctene **112** in 61% yield (Scheme 25) [49]. The difference between these two diastereoisomers, **107** and **111**, is attributed to the preferred conformation of the substrate. The introduction of the sp^2^ carbon at the C-2 position forces two olefin partners close to each other by the Thorpe-Ingold like effect. The stereochemistry was finally determined by the careful analysis of the products.

**Scheme 26:**
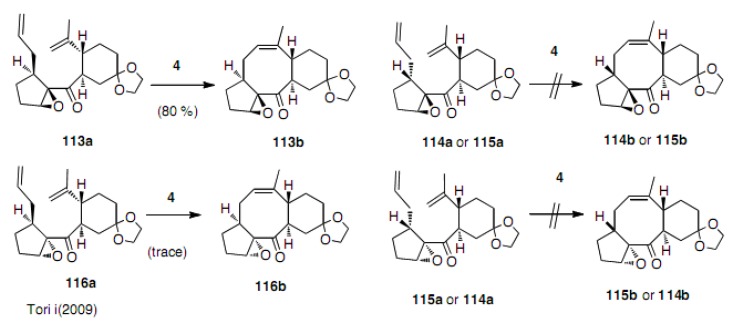
The effect of the configuration of substrates.

The four diastereoisomeric epoxides, **113a**-**116a**, were treated with the second generation Grubbs catalyst **4** to give very interesting results (Scheme 26) [50]. Among the four, only one diastereoisomer **113a** smoothly cyclized to cyclooctene derivative **113b** (80% yield), while the other three resulted in just a trace amount of cyclooctene or decomposition. This is in a good agreement with the substrate **111**. The difference in the reactivity must be due to the difference in the conformation, in which, the two ends of the olefins are in close proximity. Estimating how close they are to each other remains an unresolved issue. 

**Figure 2:**
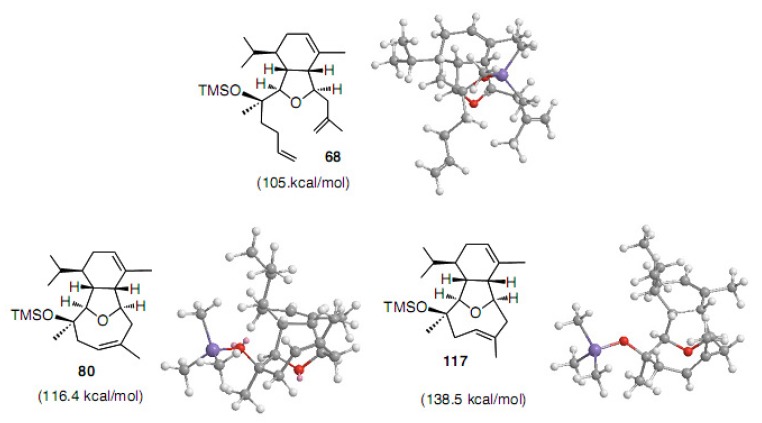

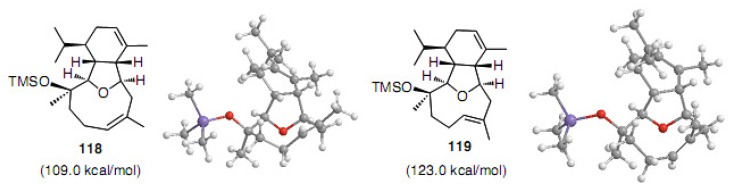
Steric energies and conformations of **68**, **80**, and **117**−**119** calculated by CONFLEX.

We have calculated the energy minimum conformation using CONFLEX [42,43] for eunicellin diterpenoid derivatives. It is quite interesting that eight-membered carbocycle **80** has a higher energy than the nine-membered carbocycle **118** with a difference of 7 kcal/mol (Figure 2). The corresponding *trans*-isomers have higher steric energies at 22−14 kcal/mol. Therefore, the cyclization reaction does not depend on the product energy, but the transition state.

**Figure 3:**
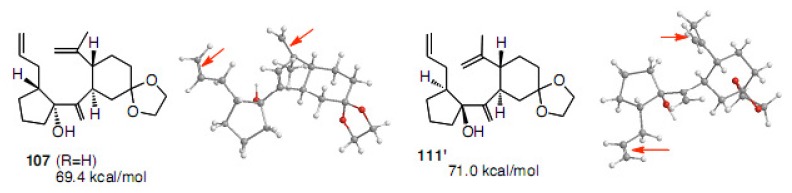
Steric energies and conformations calculated for **107** and **111'** by CONFLEX.

The stable conformations of compounds **107** and **111'** were also calculated. The steric energy for **107** is 69.4 kcal/mol and for **111'** 71.0 kcal/mol. Compound **111'** cyclized more effectively than **107**. However, the most stable conformation for **111'** was that shown in Figure 3, apparently the diene partners being distal from each other (olefins are indicated by red arrows). The cyclization does not depend on the ground state conformation, but the transition state.

**Figure 4:**
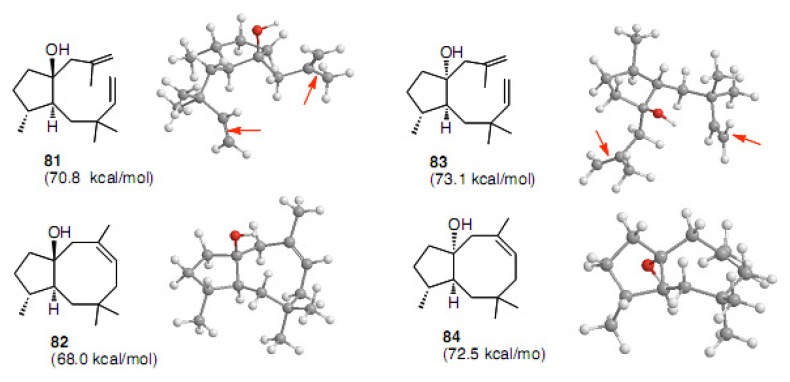
Steric energies and conformations calculated for **81**−**84** by CONFLEX.

It is also interesting that the steric energies for **82** and **84** were less than those of dienes **81** and **83**, respectively (Figure 4). These figures indicate that cyclization should occur regardless of the ring junction. The diene partners are not very far away from each other in both cases.

**Figure 5:**
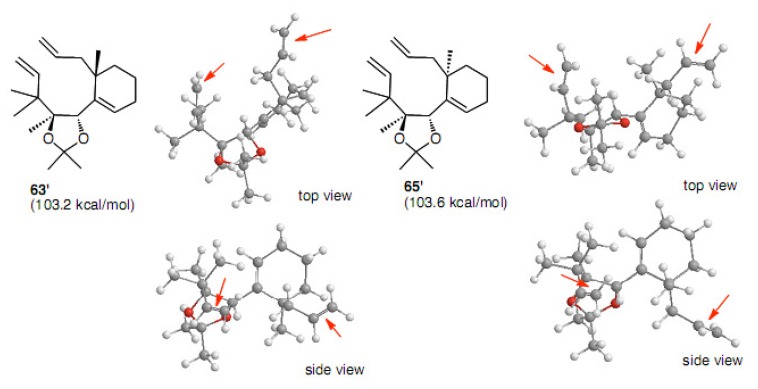
Steric energies and conformations calculated for **63'** and **65'** by CONFLEX.

The diene models of Prunet (butyl group was replaced with methyl) show that the diene partners are both in proximity, which is very interesting from the point that the substrate must be well designed and thus suitable for RCM reactions (Figure 5). They have also commented on the proximity between olefins by a brief calculation using Chem Draw [35].

## 3. Conclusions

In this review, cyclization reactions to cyclooctenes were briefly introduced, not only with trisubstituted double bonds but also with disubstituted double bonds. In a model study, only disubstituted eight-membered carbocycles are shown to be realized using catalyst **4**. A large number of examples applied in this area have been reported. However, the success by Fürstner prompted us to attempt the synthesis of trisubstituted cyclooctene using the RCM reactions introduced above. In the literature, only the successful reactions are reported, and many examples of unsuccessful reactions and the corresponding data remain undisclosed. In the successful examples, the substrates have been eloquently designed to meet the requirements of the reactions. The diene partners are most likely allowed to interact in the reaction conditions. A type of Thorpe-Ingold effect appears to force both diene ends close to each other. The length of both chains bearing the olefin moiety should be designed to be equal, because the chance of interaction increases. In this context, examples **97** and **100** failed to cyclize; however, examples **107** and **111** succeeded. In these examples the introduction of the exomethylene group greatly aided the formation of the eight-membered carbocycle. It is again important to note that the substrate must be suitably designed to meet the requirement of cyclization.
